# α/β hydrolase domain-containing protein 1 acts as a lysolipid lipase and is involved in lipid droplet formation

**DOI:** 10.1093/nsr/nwae398

**Published:** 2024-11-07

**Authors:** Ismael Torres-Romero, Bertrand Légeret, Marie Bertrand, Damien Sorigue, Alicia Damm, Stéphan Cuiné, Florian Veillet, Carla Blot, Sabine Brugière, Yohann Couté, Matthew G Garneau, Hari K Kotapati, Yi Xin, Jian Xu, Philip D Bates, Abdou R Thiam, Fred Beisson, Yonghua Li-Beisson

**Affiliations:** Aix Marseille Univ, CEA, CNRS, Institute of Bioscience and Biotechnology of Aix Marseille, BIAM, Saint-Paul-Lez-Durance 13108, France; Aix Marseille Univ, CEA, CNRS, Institute of Bioscience and Biotechnology of Aix Marseille, BIAM, Saint-Paul-Lez-Durance 13108, France; Aix Marseille Univ, CEA, CNRS, Institute of Bioscience and Biotechnology of Aix Marseille, BIAM, Saint-Paul-Lez-Durance 13108, France; Aix Marseille Univ, CEA, CNRS, Institute of Bioscience and Biotechnology of Aix Marseille, BIAM, Saint-Paul-Lez-Durance 13108, France; Laboratoire de Physique de l'École Normale Supérieure, ENS, Université PSL, CNRS, Sorbonne Université, Université de Paris Cité, Paris 75005, France; Aix Marseille Univ, CEA, CNRS, Institute of Bioscience and Biotechnology of Aix Marseille, BIAM, Saint-Paul-Lez-Durance 13108, France; Aix Marseille Univ, CEA, CNRS, Institute of Bioscience and Biotechnology of Aix Marseille, BIAM, Saint-Paul-Lez-Durance 13108, France; Aix Marseille Univ, CEA, CNRS, Institute of Bioscience and Biotechnology of Aix Marseille, BIAM, Saint-Paul-Lez-Durance 13108, France; Univ. Grenoble Alpes, INSERM, CEA, UMR BioSanté U1292, CNRS, CEA, FR2048, Grenoble 38000, France; Univ. Grenoble Alpes, INSERM, CEA, UMR BioSanté U1292, CNRS, CEA, FR2048, Grenoble 38000, France; Institute of Biological Chemistry, Washington State University, Pullman 99164, USA; Institute of Biological Chemistry, Washington State University, Pullman 99164, USA; Single-Cell Center, CAS Key Laboratory of Biofuels and Shandong Key Laboratory of Energy Genetics, Qingdao Institute of Bioenergy and Bioprocess Technology, Chinese Academy of Sciences, Qingdao 266101, China; Single-Cell Center, CAS Key Laboratory of Biofuels and Shandong Key Laboratory of Energy Genetics, Qingdao Institute of Bioenergy and Bioprocess Technology, Chinese Academy of Sciences, Qingdao 266101, China; Institute of Biological Chemistry, Washington State University, Pullman 99164, USA; Laboratoire de Physique de l'École Normale Supérieure, ENS, Université PSL, CNRS, Sorbonne Université, Université de Paris Cité, Paris 75005, France; Aix Marseille Univ, CEA, CNRS, Institute of Bioscience and Biotechnology of Aix Marseille, BIAM, Saint-Paul-Lez-Durance 13108, France; Aix Marseille Univ, CEA, CNRS, Institute of Bioscience and Biotechnology of Aix Marseille, BIAM, Saint-Paul-Lez-Durance 13108, France

**Keywords:** algae, betaine lipid, α/β hydrolase, lipid droplet, lysolipid

## Abstract

Lipid droplets (LDs) are the major sites of lipid and energy homeostasis. However, few LD biogenesis proteins have been identified. Using model microalga *Chlamydomonas*, we show that ABHD1, an α/β-hydrolase domain-containing protein, is localized to the LD surface and stimulates LD formation through two actions: one enzymatic and one structural. The knockout mutants contained similar amounts of triacylglycerols (TAG) but their LDs showed a higher content of lyso-derivatives of betaine lipid diacylglyceryl-*N,N,N*-trimethylhomoserine (DGTS). Over-expression of *ABHD1* increased LD abundance and boosted TAG content. Purified recombinant ABHD1 hydrolyzed lyso-DGTS, producing a free fatty acid and a glyceryltrimethylhomoserine. *In vitro* droplet-embedded vesicles showed that ABHD1 promoted LD emergence. Taken together, these results identify ABHD1 as a new player in LD formation by its lipase activity on lyso-DGTS and by its distinct biophysical property. This study further suggests that lipases targeted to LDs and able to act on their polar lipid coat may be interesting tools to promote LD assembly in eukaryotic cells.

## INTRODUCTION

Lipid droplets (LDs) are the site of lipid storage accumulation in eukaryotic cells. LDs are made of a neutral lipid core, consisting of mostly triacylglycerols (TAGs), and are surrounded by a monolayer of polar lipids (hemi-membrane) embedded with proteins [1]. LDs evolved to sequester the non-membrane-forming neutral lipids, i.e. TAG, away from membranes. In addition to their major role as carbon storage, LDs contribute to maintain energy homeostasis, prevent lipotoxicity, act as a temporary depot for acyl chains and participate in lipid signaling [2–5]. Thermodynamically speaking, the oil-in-water configuration of LDs sets them apart from all other subcellular organelles. Their formation therefore requires tight coordination between the biophysical properties of their neutral lipid core and amphipathic polar lipids, and those of their protein coat. Accumulating evidence points to the importance of both proteins and lipids in maintaining a thermodynamically stable LD configuration and population [6–8].

LD biogenesis occurs in response to environmental cues (such as light, nutrition and temperature) or is developmentally regulated (as in seeds or in aging cells). Considering the paramount importance of TAG metabolism for human health and for biotechnology, LD formation has been studied intensively in various organisms ranging from yeast to mammals, land plants and microalgae [3,9]. Current understanding is that the LD, at least most of it, emerges from specific subdomains of the endoplasmic reticulum (ER) membrane defined by the enrichment of various proteins including the protein SEIPIN found in mammals, plants and fungi [10]. Initial membrane lipid remodeling and TAG synthesis results in the formation of an ‘oil lens’ between the leaflets of the ER bilayer, which is then followed by its growth, and finally it pinches off the membrane toward the cytosol, giving rise to an LD [6]. It is worth pointing out that new evidence suggests that LDs may not necessarily ever pinch off from the ER, but rather remain attached to the ER [11]. Thanks to the development of affordable proteomics tools, LD proteomes of many species along the evolutionary tree are now published, pointing to the divergent nature of the proteins that are associated with LDs [12,13]. Up to now, however, only a handful of proteins have been demonstrated to play a role in LD biogenesis in plants, animals or yeast. These include oleosin, caleosin, PERILIPIN1, FSP27 (fat-specific protein 27), FIT2 (fat storage-inducing transmembrane protein 2), the FATP1-DGAT2 (fatty acid transport protein 1-diacylglycerol acyltransferase 2) complex, the LD-associated protein (LDAP), and finally SEIPIN with its interactors, i.e. LD-assembly factor 1 (LDAF), the LDAP-interacting protein (LDIP) or the membrane-tethering protein VAP27-1 (vesicle-associated protein 27–1) [1,9,14–23].

In the past 10 years, *Chlamydomonas reinhardtii* has been used as a model green microalga to interrogate LD biogenesis and turnover [2]. LD formation and degradation in *Chlamydomonas* can be manipulated easily, for example, by simply changing the nitrogen (N) level in the media [24]. N starvation is one of the most potent triggers in inducing TAG accumulation, which results in a drastic increase in both LD number and size in algal cells [24]. In addition to research on environmental conditions or the genetic basis of TAG metabolism and regulation [14,24–35], the protein and lipid composition of the LD coat has also been reported [32,36–38]. The monolayer lipid-coat of an LD found in *Chlamydomonas* is made predominantly of the betaine lipid diacylglyceryl-*N,N,N*-trimethylhomo-Ser (DGTS), followed by sulfoquinovosyl diacylglycerol (SQDG) and phosphatidylethanolamine (PE) and small amounts of galactolipids [32]. Over 200 proteins were found in an LD-enriched fraction in *Chlamydomonas* [37], but only a few have been characterized. This includes: the major lipid droplet protein (MLDP), considered a structural protein of *Chlamydomonas* LDs [36,39], the betaine lipid synthase BTA1 [40], the long-chain acyl activating enzyme LCS2 [41,42] and various lipid trafficking proteins [43]. No protein involved in LD budding and expansion has been identified yet in algae.

To investigate LD biogenesis in *Chlamydomonas*, we have focused on a putative α/β hydrolase domain-containing protein (ABHD1), which is present in the proteomics of LDs isolated from N-starved and high light-exposed cells [32,37]. Using cell biological, biophysical, genetic (gain- and loss-of-function) and biochemical studies in *Chlamydomonas*, we demonstrate here that ABHD1 is a lipase mostly acting on lyso-DGTS that can promote LD formation *in vivo* and *in vitro*. A tentative model describing the dual role—enzymatic and non-enzymatic—of ABHD1 in the mechanism of LD formation is also proposed.

## RESULTS

### Gene expression and structural and evolutionary features of ABHD1 protein

**Figure 1.**
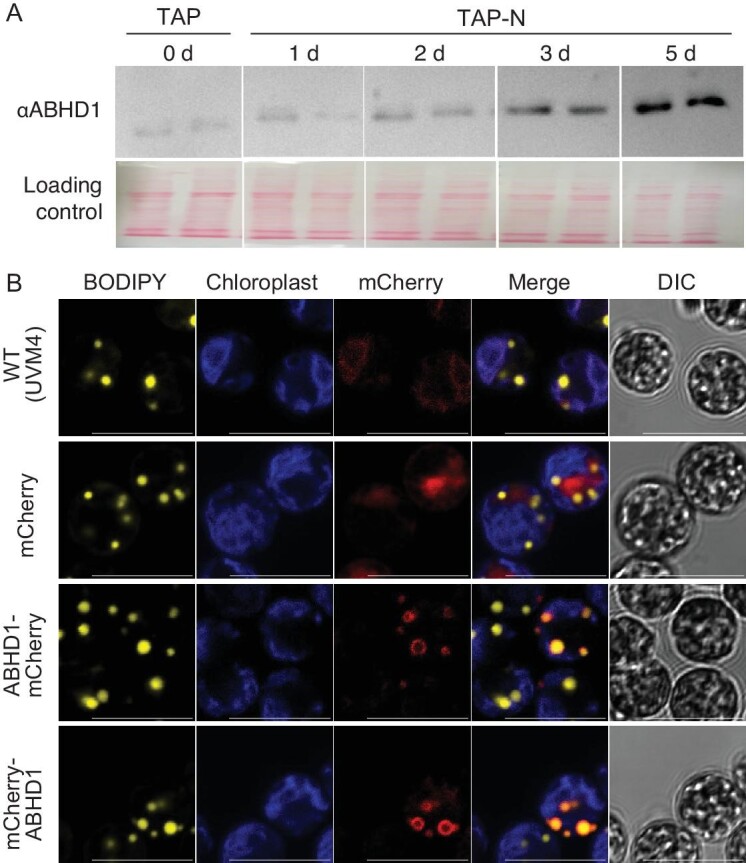
ABHD1 protein is located on the surface of LDs and its quantity increases with prolonged N starvation. (A) ABHD1 protein level as detected by immunoblot analysis using anti-ABHD1 antibodies. Total protein extracts (from 0.4 million cells) were loaded onto each lane. Ponceau's stained proteins transferred onto nitrocellulose membrane were shown as the control for protein load (image height of whole lane was compressed). Abbreviations: α, antibody; d, day; N, nitrogen; TAP, tris-acetate-phosphate media. (B) Confocal imaging. Cells were cultured in TAP-N (1 d) and stained with BODIPY to observe LDs. Pseudo-colors were used: BODIPY-stained LDs in yellow, chlorophyll autofluorescence in blue, mCherry and mCherry-tagged ABHD1 in red. DIC: differential interference contrast. Bar = 10 µm.

Searching public databases on mRNA expression levels for *ABHD1*, one can observe that its gene expression is increased 2-fold upon N starvation [44]. Furthermore, our immunoblot using anti-ABHD1 antibodies showed that ABHD1 significantly increased in *Chlamydomonas* cells during N deprivation, a condition promoting TAG synthesis and LD formation (Fig. 1A, Fig. S1). Taken together, these data strongly suggest that ABHD1 may play a role in LD formation in *Chlamydomonas*. In addition to ABHD1, the *Chlamydomonas* genome encodes another protein (Cre01.g010550) showing 51% sequence identity to ABHD1, which we named ABHD2. ABHD2 is absent in the three previously published LD proteomics for *Chlamydomonas* either isolated from N-starved cells or after high light exposure [32,45,46], and moreover its expression is not regulated by N deprivation [37,44], therefore it is very unlikely that ABHD2 plays a complementary role in terms of LD biology.

To gain insight into ABHD1 protein functions, we examined its primary amino acid sequence, its hydrophobicity [47] and its predicted structural features using alpha-fold [48]. ABHD1 is composed of three major parts: an N-terminal transmembrane domain, one central α/β-hydrolase fold-containing domain, and an intrinsically disordered domain at its C-terminus (Fig. S2A–C). In addition, the occurrence of a hydrophobic helix (alanine-rich domain) at its C-terminus (AA 396–490) (Fig. S2B and C) could possibly serve as an anchor to LDs. Similar structural motifs at the C-terminus of the phosphatidylserine-specific phospholipase A1 have been shown to bind lipids [49]. In terms of hydrophobicity (Fig. S2E), ABHD1 shares similar overall hydrophobic features to MLDP but is a bit less hydrophobic (Gravy index of 0.05 compared to 0.11 for MLDP) and does not contain the major hairpin hydrophobic domain present in oleosins [36].

A phylogenetic tree based on an amino acid sequence was constructed to explore the evolutionary history of ABHD1, which included 92 representative proteins from different clades of life (Table S1). The closest homologs to ABHD1 were grouped in a clade distinct from bacterial and cyanobacterial homologues and this clade only included algae of the Chlorophytina taxon, which is composed of the classes Ulvophyceae, Trebouxiophyceae and Chlorophyceae (Fig. S3). ABHD1 homologs are absent in plant or mammalian cells, therefore ABHD1 is specific to Chlorophyta. The lack of homology in these different lineages seems to have been a rule rather than an exception for LD-associated proteins such as, for example, the protein Delayed in TAG Hydrolysis 1 (DTH1) [35]. Increasing evidence seems to point out that organisms from distinct lineages equip their LDs with unique sets of proteins that do not share primary sequence homology but follow the overarching principles in LD structure/architecture and function.

### ABHD1 coats the entire LD and its over-expression induces LD formation

To investigate the functional role of ABHD1, we first sought to confirm that the protein was actually associated with LDs *in vivo* and determine whether it is its major or only location. We therefore fused the fluorescent protein mCherry at the N- or C-terminus of the ABHD1 protein (Fig. S4A, Table S2) and transformed the nuclear genome of *Chlamydomonas* with a construct expressing this chimeric protein under the strong constitutive photosystem I protein D (PSAD) promoter. To facilitate LD identification under the confocal microscope, N-deprived cells were examined and stained with the lipid dye BODIPY. More than 500 antibiotic-resistant *Chlamydomonas* transformants (both N- and C-terminal fusions) were screened based on mCherry signal. The mCherry signal was detected all around LDs, regardless of the N- or C-terminal position of the tag (Fig. 1B, Figs S5 and S6, Movie S1). Nevertheless, a higher number of positive lines were obtained when mCherry was fused to the N-terminus of ABHD1 protein. The fusion of mCherry to the C-terminus of ABHD1 seems to be less stable, leading to a higher rate of truncated or misfolded chimeric protein. If the disordered C-terminus region is important for proper folding and 3D structure or targeting to LDs, this could explain the lower amounts of over-expressors (OEs) identified (Fig. S2B). We have therefore focused on the lines where mCherry is fused to the N-terminus of the ABHD1 protein to minimize misfolded proteins, such as aggregates or mis-targeted protein, which could occasionally be observed via mCherry signal in the cytosol. A more likely reason for mCherry signal in the cytosol is the use of the strong PSAD promoter, which resulted in potential ABHD1 over-expression. The robust signal emitted by mCherry fluorescence around the LD surface strongly indicates that ABHD1 is essentially a LD-associated protein and coats the entire LD (Movie S1).

**Figure 2.**
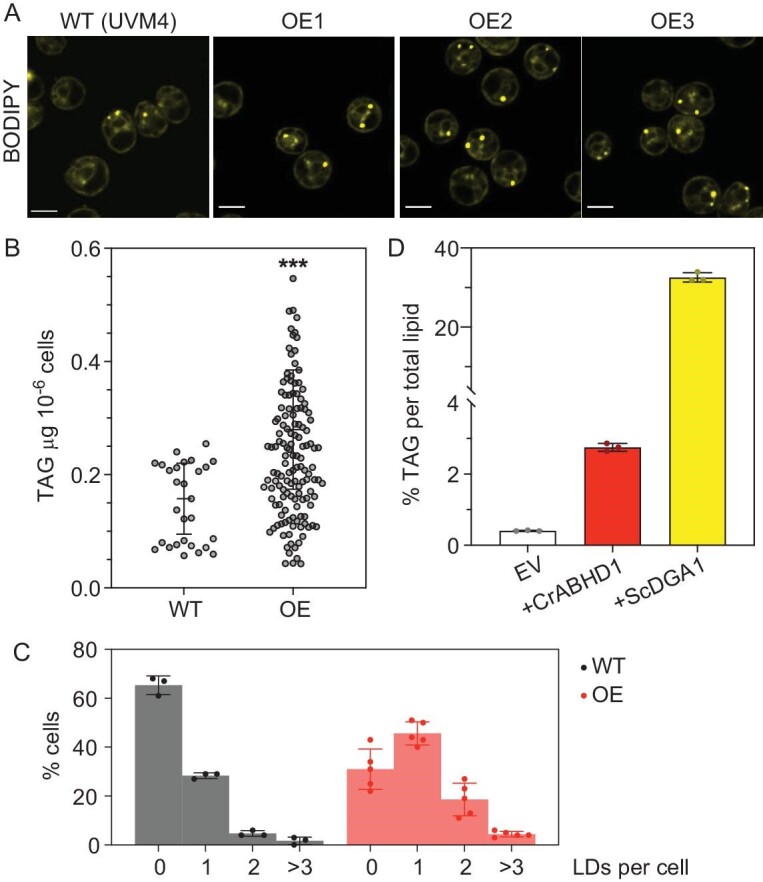
*ABHD1* over-expression results in LD formation and a higher TAG content. (A) LD imaging in cells over-expressing *ABHD1* in nitrogen-replete mixotrophic growth, i.e. TAP medium (three independent lines are shown, bar = 5 µm). (B) TAG amount in WT versus *ABHD1* over-expressing lines during nitrogen replete mixotrophic growth. Four independent experiments, for six independent lines (three technical replicates for each line), are shown. Error bars show mean and standard deviation, Mann-Whitney test: ***P = 0.0002. (C) The distribution of LD numbers per cell during nitrogen replete mixotrophic growth (>200 cells examined per group). Histograms represent mean and standard deviation of three biological replicates for WT and five independent lines for OE (*ABHD1* overexpressor). (D) TAG quantification in the H1246 yeast strain expressing the *Chlamydomonas ABHD1* gene. Yeast transformants expressing the respective gene were harvested and analyzed for total TAG amount using gas chromatography–mass spectrometry (GC-MS). Data are means of three biological replicates with standard deviation bars shown. A significance of *q* = 0.13 for empty vector (EV) vs. +CrABHD1, *q* = 0.02 for EV vs. +ScDGA1 and *q* = 0.13 for +ScDGA1 vs. +CrABHD1 was determined using the Kruskal–Wallis test followed by a two-stage step-up Benjamini, Krieger and Yekutieli false discovery rate procedure for multiple comparison correction.

While performing the subcellular localization experiment, we noticed LDs seem to be more abundant in *ABHD1* overexpressing lines during optimal growth than in the control parental line i.e. UVM4 cells (Fig. 2A). Lipid analysis revealed that, while there was no difference in TAG content during N starvation between the *ABHD1* OEs and the parental strain UVM4 (Fig. S4B), OEs contained more TAGs during N replete growth (Fig. 2B) without significant difference in TAG composition (Fig. S4C). Interestingly, the number of LDs per cell was also altered, with a shift toward a higher number of LDs per cell in the OEs compared to the parental line (Fig. 2C). These results thus indicate that in *Chlamydomonas* ABHD1 is not a limiting factor for LD accumulation under N-depletion but can induce the formation of LDs, or block LDs’ degradation under N-replete conditions when over-expressed. In order to see whether ABHD1 could induce LD formation in cells other than a microalga, we also expressed *ABHD1* in the quadruple yeast mutant H1246 strain where TAG synthesis is greatly reduced. The yeast H1246 strain is knocked out in genes encoding a diacylglycerol acyltransferase1 (DGA1), lecithin cholesterol acyltransferase (LRO1) and acyl-coenzyme A: cholesterol acyl transferase-related enzyme 1 and 2 (ARE1 and ARE2). It has greatly reduced but not nulled TAG synthesizing activity [50]. *Chlamydomonas ABHD1* (*CrABHD1*) expression alone in the *Saccharomyces cerevisiae* H1246 mutant resulted in a significant increase in TAG compared to the vector control line (Fig. 2D). This increase in TAG was not as dramatic as with the native *S. cerevisiae* TAG-synthesizing enzyme diacylglycerol acyltransferase 1 (ScDGA1), but it was nevertheless not negligible. This result suggests that a similar role of ABHD1 either in LD induction or in preventing LD turnover/TAG degradation exists in yeast. It also rules out that the observed TAG increase in *Chlamydomonas* ABHD1 OEs is due to the presence of the mCherry tag.

### Absence of ABHD1 affects the composition of the LD coat

**Figure 3.**
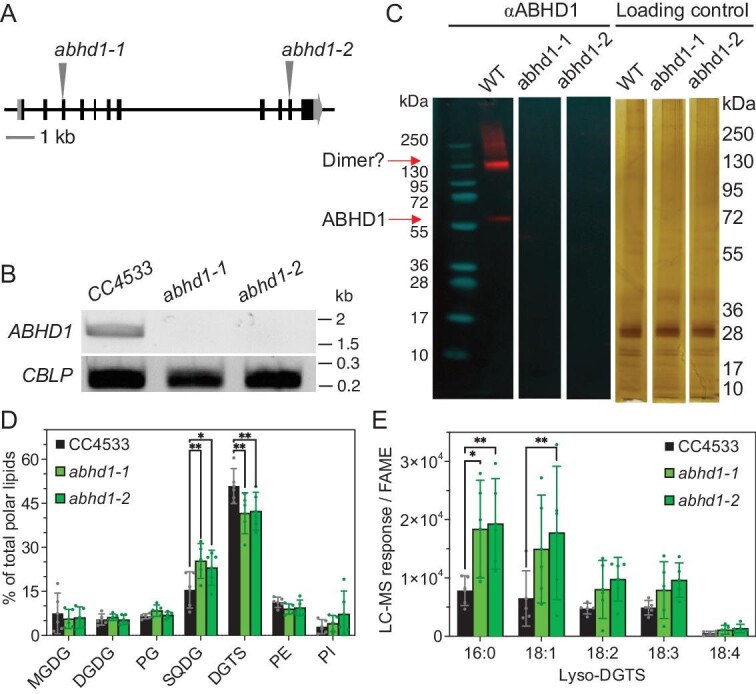
Isolation and characterization of lipids for the two *abhd1-1* and *abhd1-2* mutants. (A) The site of the *AphVIII* cassette insertion in the *ABHD1* gene for *abhd1-1* and *abhd1-2 mutants*. (B) RT-PCR analysis of *ABHD1* transcript. (C) Immunoblot analysis of ABHD1 protein on isolated LDs from the three strains. LDs were extracted from cells N-starved for 2 d. Proteins were extracted then loaded into each lane, based on a fixed number of fatty acid methyl esters (FAMEs) (i.e. ca. 55 μg FAME equivalent of LDs loaded). Silver-stained SDS-PAGE of LD-isolated proteins acts as a loading control. (D) Membrane coat composition of isolated LDs from nitrogen starved cultures. Levels of lyso-DGTS species detected in the polar lipid fraction of isolated LDs. Signal from ultra-high performance liquid chromatography with quadrupole time-of-flight mass spectrometry (LC-MS) was normalized by FAME quantities in the polar lipid fraction. (E) Changes in LD lyso-DGTS species in the mutants in comparison to WT during nitrogen starvation. For both (D) and (E): data are means of five biological replicates from three independent LD isolations. Bars indicate standard deviation. *P* values were determined, after passing Bartlett's test for homoscedasticity and Shapiro-Wilk's test for normality, by a two-way ANOVA with Bonferroni correction: *P < 0.05; **P < 0.01. Abbreviations: CBLP: *Chlamydomonas* beta subunit-like polypeptide; FAME, fatty acid methyl ester; MGDG, monogalactosyldiacylglycerol; DGDG, digalactosyldiacylglycerol; PG, phosphatidylglycerol; SQDG, sulfoquinovosyldiacylglycerol; DGTS, diacylglyceryl-*N,N,N*-trimethylhomoserine; PE, phosphatidylethanolamine; PI, phosphatidylinositol.

To further investigate the function of ABHD1 in LD biogenesis, we isolated two insertional mutants from the *Chlamydomonas* mutant library (CLIP) [41]. The two independent lines *abhd1-1* and *abhd1-2* harbor an insertion at the 3rd and 10th exon, respectively (Fig. 3A). Using gene specific primers, reverse transcription-polymerase chain reaction (RT-PCR) analyses showed that there was no *ABHD1* expression in *abhd1-1* and *abhd1-2* mutant alleles (Fig. 3B). Immunoblot analyses of total proteins extracted from isolated LDs from respective WT and the two mutant strains showed that no ABHD1 protein could be detected in either mutant (Fig. 3C). Interestingly, the immunoblot from WT cells indicated that ABHD1 could be present as a dimer. Taken together, both gene expression and immunoblot analyses firmly confirmed both alleles as true knockout mutants. We then compared the TAG amount in the two mutants (*abhd1-1* and *abhd1-2*) to their parental strain (i.e. CC4533). No difference in TAG content was observed under N-replete or N-deplete conditions (Fig. S7A and C). Whole-cell lipidomics showed no significant change in TAG composition nor membrane lipids either (Fig. S7B, D, E). LD number and size are also similar between CC4533 and the two knockout mutants (Fig. S7F and G).

To investigate further the role that ABHD1 may have in LD formation *in vivo*, we performed comparative lipidomics and proteomics of isolated LDs from N-starved WT and the two knockout mutants. DGTS was identified as a major component of LD lipid-coat, followed by SQDG and PE. The mutants’ LD coat showed a remodeled lipid composition (Fig. 3D), with a lower proportion of the major lipid class (DGTS) but a higher proportion of SQDG, a class of negatively charged lipids. Among the lipid species with the most significant differences were some lyso-DGTS species, in particular lyso-DGTS 16 : 0, which were present in significantly higher amounts in the mutants’ LD coat compared to WT (Fig. 3E).

To determine whether the mutants affected the flux of nascent fatty acids through membrane lipids into TAG during N-starvation, we performed a [^14^C]acetate pulse-chase analysis (Fig. S8). There were limited differences in the precursor–product relationships of membrane lipids and TAG between WT, *abhd1* mutants and the over-expressing lines, indicating that the action of ABHD1 does not significantly affect the major flux of acyl chains into TAG during N-starvation.

We explored potential proteomics changes in the isolated LDs (Fig. S9, Data Set S1). Among the 598 proteins repeatedly detected by mass spectrometry (MS)-based proteomics, only four showed a significant change to the relative amount between the WT and the two mutants (Data Set S1). These four proteins were reduced in the knockout mutant LDs: ABHD1 (as expected), the long-chain acyl-CoA synthetase (LCS2), the photosystem I reaction center subunit H (PSAH), and a putative glycosyl hydrolase (GHL1). The reduction in the LCS2 protein amount in the two knockout mutants supports the idea that in the mutant there is indeed a reduced need for acyl-activation, corroborating the enzymology data below showing that ABHD1 is a lipase (next section). GHL1 has been reported to potentially function as a galactolipid galactosyl hydrolase and provide DAG for TAG synthesis [51], but it remains to be tested whether GHL1 participates in glyceryl-*N,N,N*-trimethylhomoserine (GTS) hydrolysis, allowing, therefore, glycerol recycling downstream of ABHD1. Taken together, these data suggest that ABHD1 may act in the modification of the monolayer of the LD coat, as its absence results in changes in both LD membrane lipid and protein composition.

### ABHD1 has lyso-DGTS lipase activity

**Figure 4.**
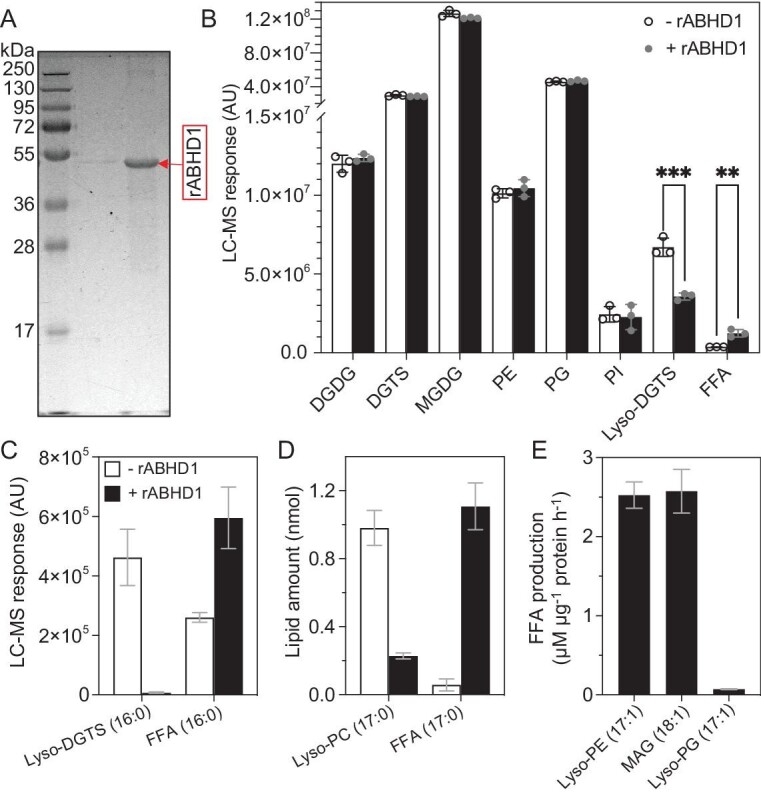
Enzyme activity analysis of the refolded rABHD1 protein. (A) SDS-PAGE of rABHD1 purified from *E. coli* inclusions bodies and refolded. (B) Activity test of rABHD1 on *abhd1-1* total lipid extracts identified lyso-DGTS as potential ABHD1 substrate. LC-MS detected over 10 000 m/z peaks for both the reactions with and without rABHD1. The full spectrum from LC-MS is provided in Fig. S10. (C) The lipase activity of rABHD1 toward purified lyso-DGTS. (D) The lipase activity of rABHD1 toward commercial lyso-PC. (E) Activity assay on lyso-PE, lyso-PG, MAG, PC and DGTS. But, no activity could be detected on PC or on DGTS for rABHD1. Data are the mean of three independent experiments and error bars refer to standard deviation. Student's t-test is applied after passing Bartlett's test for homoscedasticity and Shapiro-Wilk's test for normality: **P < 0.01, ***P < 0.001. Free fatty acid (FFA) formation was quantified using 19:0 fatty acid as an internal standard. Reaction conditions for all activity tests: Teorell Stenhagen universal buffer pH 7.5, with 100 mM NaCl and Triton-X100 at 1 CMC. During the enzymatic reaction (incubation of 2 h), during the preparation of lysolipid substrate or during lipid extraction, there is always some autolysis of the substrate, which is responsible for the presence of a certain amount of free fatty acids in the negative control.

To investigate the potential enzymatic activity of ABHD1, we expressed in *Escherichia coli* a truncated version of ABHD1 lacking the N-terminal hydrophobic portion (with amino acids 1 to 46 removed) resulting in recombinant ABHD1 (rABHD1). Purification of the soluble native rABHD1 was only partial but, in the presence of urea, a highly pure rABHD1 protein was obtained from inclusion bodies and refolded (Fig. 4A, Table S3). To identify its potential substrate(s), the refolded purified rABHD1 was first incubated with total lipid extracts of *Chlamydomonas abhd1-1* mutant. Interestingly, we observed a reduction in lyso-DGTS species and an increase in free fatty acids, but no change in the other major lipid molecular species (Fig. 4B, Fig. S10A). We could also detect changes in some other very minor lysolipid species (such as lyso-MGDG, lyso-SQDG, lyso-PG and lyso-PE) but not lyso-DGDG (Fig. S10B). These other lysolipids were not further tested because they are >100 times less abundant than lyso-DGTS. ABHD1 activity was then tested using pure lyso-DGTS as substrate, prepared from the partial spontaneous hydrolysis of commercial DGTS. Results showed that ABHD1 indeed acted as a lipase on pure lyso-DGTS, as the two expected co-products of the reaction (Fig. 4C), palmitic acid and GTS, could be identified by MS (Fig. S11).

Similar activity could be obtained using lyso-phosphatidylcholine (lyso-PC), a commercially available lipid structurally similar to lyso-DGTS (Fig. 4D). Due to the difficulty in obtaining purified lyso-DGTS, further characterization of the putative enzymatic activity of rABHD1 was carried out on lyso-PC. Activity assays conducted under various conditions of buffer, pH, detergent or cations (Fig. S12) allowed us to select a reaction medium for further studies. Release of free fatty acid from lyso-PC was found to increase with time and with rABHD1 amount, which showed that it corresponded to genuine enzymatic activity (Fig. S13).

ABHD1 did not show activity toward lipids with two acyl-groups (Fig. 4B, Table S4), therefore we tested the activity of rABHD1 toward other lipids containing one fatty acid, e.g. lyso-phosphatidylethanolamine (lyso-PE), lyso-phosphatidylglycerol (lyso-PG) and monoacylglycerol (MAG). Results showed that rABHD1 protein released fatty acids from lyso-PE, MAG and lyso-PG but showed relatively low activity toward the latter (Fig. 4E). This was observed not only when incubated with total lipid extracts, but also purified commercial substrates as well (Fig. 4D and E). These results thus indicated that rABHD1 has some preference for lysolipids over diacyl-lipid analogs (no activity toward lipids with two acyl-groups as shown in Fig. 4B), and it did not show any acyltransferase or transacylase activity (Table S4).

### ABHD1 favors LD budding *in vitro*

**Figure 5.**
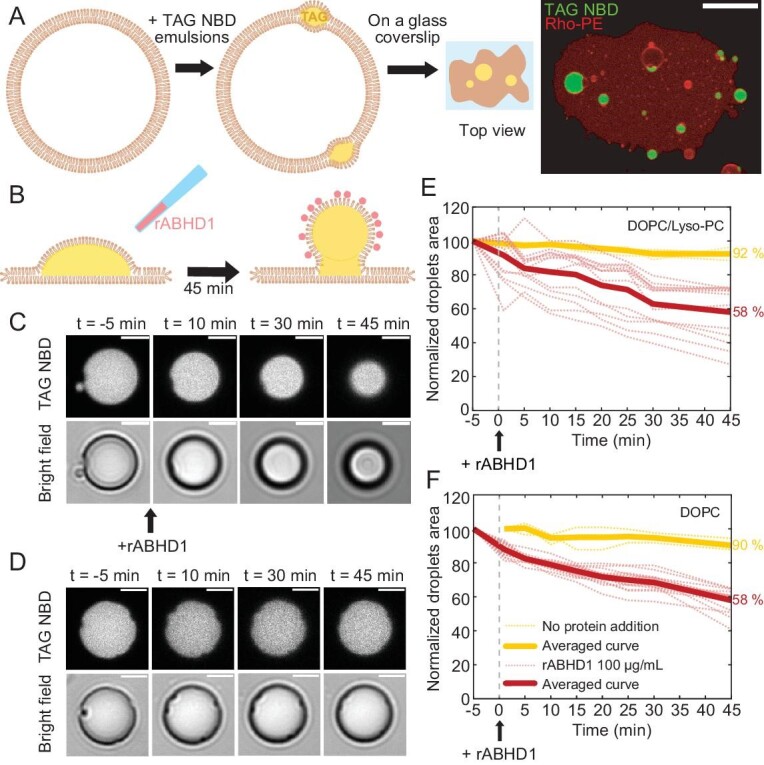
Addition of the rABHD1 protein to the GUV boosts curvature formation. (A) Schematic view of the experimental protocol. GUVs are put in contact with a thin emulsion of NBD-labeled triacylglycerol (TAG) that forms droplets at the lipid bilayer. When placed on a glass coverslip, membrane rupture results in a 2D lipid bilayer at the surface with droplets of TAG incorporated in. Right: typical image of a DEV after rupture is shown with Rho-PE in red and TAG NBD in green. Scale bar is 10 µm. (B) The droplet initially forms a spherical cap and is budding from the membrane 45 min after rABHD1 addition in the observation buffer (100 µg mL^−1^). (C) Time lapse of a TAG droplet at the membrane plane (NBD TAG signal in upper panel and bright field in lower panel), showing the decrease of the surface after addition of rABHD1. Scale bars are 2 µm. (D) Control time lapse of a TAG droplet at the membrane plane without addition of protein. Scale bars are 2 µm. (E, F) Measurement of the droplet surface at the membrane plane according to time in two different lipid compositions: (E) DOPC/Lyso-PC/RhoPE and (F) DOPC/RhoPE. The measured area of each droplet is normalized by the initial surface occupied at t = −5 min. Dashed lines represent the time traces of individual droplets and thick lines are the average trend. Light yellow lines correspond to the control in the absence of ABHD1 [(E) N = 3 droplets analyzed, (F) N = 4], and dark red lines to the addition of rABHD1 at 100 µg mL^−1^ [(E) N = 16, (F) N = 13].

We further investigated the effect of ABHD1 association and lysolipid hydrolysis on the geometry of LDs. To do so, we employed the droplet-embedded vesicle (DEV) system [52,53]. Model LDs are incorporated into pre-formed giant unilamellar vesicles (GUVs) producing DEVs (Fig. 5A). We then deposited DEVs on a glass coverslip which caused them to rupture (Fig. 5B), resulting in a 2D bilayer with the droplets incorporated [54]. Resulting LDs were immobilized and their geometry was followed throughout. The phospholipid composition was as follows: dioleoyl phosphatidylcholine (DOPC)/lyso-PC/rhodamine-PE (79.5/20/0.5% w/w), and TAG to form neutral LDs. Fluorescent lipid rhodamine-PE acted as a membrane reporter and nitrobenzoxadiazole (NBD)-labeled TAG allowed us to visualize the LD. The model LDs’ initial shape is a spherical cap, and addition of the purified rABHD1 resulted in the transition of the model LD from a flattened to a budded shape. This transition is shown by the fact that in the lipid bilayer the projected droplet surface area decreased with time by 40% as compared to the control, i.e. without any protein added (Fig. 5C and D). Moreover, we obtained similar results with pure DOPC DEVs, i.e. in the absence of lyso-PC, the major substrates of ABHD1 (Fig. 5E and F). Furthermore, when a non-refolded urea-purified form of rABHD1, which was enzymatically completely inactive (see Material and Methods), was tested in the DEV system, budding was still observed despite having slightly weaker capacity in comparison to the wild-type version of the protein (Fig. S14A). The fact that the denatured rABHD1 had weaker budding than the refolded rABHD1 may be due to the harsh denaturation conditions used, which may have impacted negatively (directly or indirectly) the rABHD1 domain involved in the budding. Addition of bovine serum albumin (BSA) instead of ABHD1 protein did not result in LD budding, providing further support that the above observation is specific to ABHD1 (Fig. S14B). Taken together, the above two lines of evidence clearly show that, independently of its lyso-DGTS lipase function, the binding of ABHD1 to the model LDs’ surface participates in droplet budding [52].

## DISCUSSION

**Figure 6.**
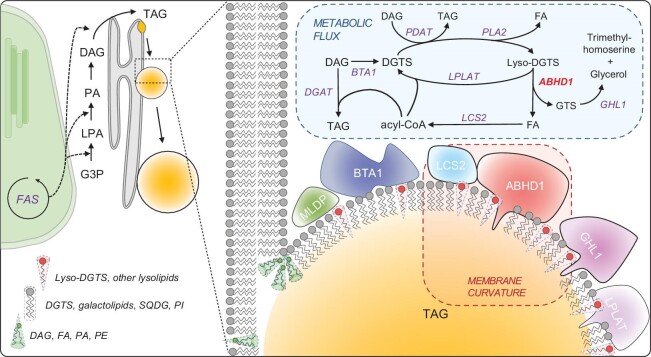
A model explaining the multifaceted actions of lyso-DGTS and ABHD1 protein in promoting LD budding, growth and TAG synthesis. ABHD1 catalyzes the hydrolysis of lyso-DGTS to produce a free fatty acid and a GTS moiety. ABHD1 is present at basal levels during optimal growth, and it increases in transcription and protein amount as N starvation is initiated, paralleling the increases in both LD number and size [67]. During LD budding, the formation of lyso-DGTS and the synthesis of ABHD1 protein both promote positive membrane curvature and therefore LD budding. Later on, ABHD1 activity may facilitate LD growth as less curvature is needed by degrading lyso-DGTS. The resulting fatty acid (FA) can be activated to acyl-CoA by LCS2 and contribute to TAG synthesis. Additionally, by consuming lyso-DGTS, ABHD1 is pulling the flow of acyls from either a putative PDAT or a PLA2, whose activity drives either TAG or FA production, respectively. Indeed, in two knockout mutants, LCS2 protein is present in a reduced amount, likely because the flow of free FA is reduced without ABHD1. Taken together, the major role of ABHD1 lies in LD coat remodeling and maintaining LD stability. Abbreviations: BTA1, betaine lipid synthase 1; DAG, diacylglycerol; DGAT, diacylglycerol acyltransferase; DGTS, diacylglyceryl-3-O-4'-(*N,N,N*-trimethyl)-homoserine; FAS, fatty acid synthase; G3P, glycerol-3-phosphate; GPAT, glycerol-3-phosphate acyltransferase; MLDP, major lipid droplet protein; LCS2, long chain acyl-CoA synthetase; Lyso-PA, lysophosphatidic acid; LPLAT, lysophospholipid acyltransferase; PA, phosphatidic acid; PDAT, phospholipid: diacylglycerol acyltransferase; PLA2, phospholipase A2; TAG, triacylglycerol.

Although the proteome of *Chlamydomonas* LDs was published more than 10 years ago [36,37], only a few LD-associated proteins have been studied in any detail, and none have a demonstrated role in LD biogenesis. Here, using a combination of biophysical, cell-biological, genetic and lipidomic approaches, we show that the α/β hydrolase domain-containing protein ABHD1 acts as a lysolipid lipase and participates in LD biogenesis. Thus far, some lysolipid acyltransferases have been studied in algae, but no lysolipid lipase has yet been identified. We demonstrate here that ABHD1 catalyzes the hydrolysis of an acyl group from a lyso-DGTS molecule, and it is therefore a characterization of a new enzyme acting as a lyso-DGTS lipase, and the first lysolipid lipase identified among algae. Our current working model is that ABHD1 promotes LD emergence likely through two actions at the LD surface: a lyso-DGTS lipase activity, and a distinct non-enzymatic property that promotes LD emergence, probably altering the metabolic fate of DGTS. We provide a model of molecular events involving ABHD1 that may lead to LD emergence (Fig. 6) and we then discuss the possible implications of our findings for DGTS and TAG metabolism.

### ABHD1 is a lyso-DGTS lipase acting on the LD surface

ABHD1 belongs to the α/β-hydrolase fold domain-containing superfamily, one of the largest protein superfamilies, present in virtually all sequenced genomes [55]. Despite their large number and wide occurrence, the physiological substrates and products for members of the ABHD family remain largely uncharacterized [55]. Here, through *in vivo* and *in vitro* experiments, we provide firm evidence that ABHD1 is located at the LD surface (Fig. 1) and is a novel type of lipase, namely a lyso-DGTS lipase (Fig. 3). ABHD1 is indeed shown to be active *in vitro* on lyso-glycerolipids, including lyso-DGTS, but not on diacyl-glycerolipids such as DGTS. Similar to some other members of its superfamily, ABHD1 contains the lipase catalytic triad, i.e. Asp179, Asp328 and His356 (Fig. S2C). Furthermore, the orientation of the protein on the LD surface could be favored by the C-terminal hydrophobic helix (Fig. S2B), bringing the putative active site of the enzyme closer to the LD surface.

### Lysolipids and biophysical properties of ABHD1 contribute to LD budding

Independent of its enzymatic function, ABHD1 is also found to play a structural role in favoring LD budding and growth based on both *in vivo* and *in vitro* experiments (Figs 2 and 5). Lipidomics of isolated LDs from the two knock-out mutants show an increase in lyso-DGTS. Lysolipids contain a big polar head and only one acyl chain, and in general are present as hexagonal tubes I (H_I_) and form positively curved monolayers [56,57]. This property could be important for the budding process because positive curvature forms sharp angles. Indeed, a higher amount of lyso-DGTS was present in LDs of the two mutants. We therefore propose that at the initiation of LD formation, DGTS is converted to a lyso-DGTS, which is important for the facilitation of membrane budding. In addition, a new study has recently reported that lyso-DGTS enhances the activity of PON1 (Paraoxonase 1), and by so doing prevents oxidation of low-density lipoproteins (LDL) [58]. This study therefore points out an additional role for lyso-DGTS, i.e. boosting enzyme activity by perhaps changing LD biophysics.

Nevertheless, the accumulation of lysolipids is deleterious to the ER membrane integrity [59]. Therefore, while the generation of lyso-DGTS from DGTS may facilitate LD nucleation and budding, it will be crucial to prevent the accumulation of lysolipids. This could be done by balancing the actions of DGTS-to-lyso-DGTS conversion and lyso-DGTS degradation (Fig. 6). ABHD1 may mediate the specific degradation of lyso-DGTS (and possibly other lysolipids of the LD) by releasing free fatty acids from lysolipids. These fatty acids are then possibly used to make TAGs. A question remains as to why this reaction occurs on LDs: very likely because the LD surface offers more accessibility to lipids, as it has more packing defects [8,60], which might grant better access to lyso-DGTS and their hydrolysis/detoxification. Knowing that the ER and LDs are in contiguity, lysolipids can diffuse between these organelles, and their degradation at LDs would behave as a thermodynamic pump, driving lyso-DGTS from the ER-to-LDs for their continuous degradation.

### DGTS metabolism and TAG synthesis at the LD surface

*Chlamydomonas* lacks PC and contains instead the betaine lipid DGTS as its major extra-chloroplast membrane lipid covering the LD surface [37]. Compared to the large variety of proteins known to be involved in acylglycerol lipid metabolism, only one protein (i.e. BTA1) of betaine lipid metabolism has so far been studied, first in *Rhodobacter sphaeroides* [61], then in *Chlamydomonas* [40], and recently in *Nannochloropsis oceanica* [62]. BTA1 catalyzes the addition of S-adenosylmethionine to diacylglycerol (DAG) forming DGTS [40]. Recently, it has been shown that DGTS remodeling contributes to TAG synthesis during ER stress and this process is regulated by the transcription factor bZIP1 in *Chlamydomonas* [25]. Pulse-chase labeling experiments revealed that DGTS turnover could provide acyl groups for TAG biosynthesis, however at a much lower level than MGDG or DGDG, similar to other previous results in *Chlamydomonas* [63,64]. This explains the lack of flux of ^14^C-acyl groups from DGTS to TAG in the *abhd1* mutants, consistent with the finding that its role is not in the major TAG biosynthetic pathway, but rather in the DGTS remodeling at the LD surface. Interestingly, BTA1 together with ABHD1 is found to be among the top 10 most abundant proteins in LDs from WT cells (Data Set S1), indicating that the LD surface provides a platform for active DGTS synthesis and turnover that may be separated from the roles of DGTS in the ER membrane. The role of ABHD1 in LD coat remodeling and TAG synthesis is further supported by the changes in protein compositions observed in the two knock-out mutants (Fig. 6).

Taken together, the synthesis and degradation of lyso-DGTS at the LD surface indicates that ABHD1 plays a dual role to initiate LD formation and ensure LD growth, important in adapting membrane biophysical properties as well as in supplying acyl-chains for TAG synthesis. The fact that the knock-out mutants (*abhd1-1* or *abhd1-2*) did not show differences in their TAG content suggests that ABHD1 does not constitute a major flux in supplying acyl-chains for TAG synthesis, but rather it is important in membrane lipid coat remodeling and in preparation of biophysical membrane properties essential for LD emergence and assembly. Over-expression of the ABHD1 protein during N-replete optimal growth boosted LD formation and oil content in *Chlamydomonas* but also in yeast (Fig. 2), suggesting that ABHD1 could be used to increase TAG content of microalgae, and possibly other organisms, without compromising/halting cell growth.

## MATERIALS AND METHODS

### Strains and culture conditions

All *Chlamydomonas* strains were maintained in agar plates containing Tris-Acetate-Phosphate (TAP) media [65] under constant light at 25°C. In the case of mutants, TAP agar plates were supplemented with 15 μg mL^−1^ hygromycin or 10 μg mL^−1^ paromomycin as appropriate. Cell cultures were grown in TAP liquid media in conical glass flasks kept in incubators (Multitron, Infors HT) shaking at 120 rpm, 25°C, under continuous light (80–100 µmol photons m^−2^ s^−1^). Cell concentration, size and volume were quantified with a Multisizer 4 (Beckman Coulter). To start N deprivation, log-phase cultures were washed with fresh media without N (NH_4_Cl replaced by an equal molar amount of NaCl) at 450 *g* for 3 min.

## Supplementary Material

nwae398_Supplemental_Files

## Data Availability

All data are available in the main text or the supplementary materials. The MS proteomics data have been deposited in the ProteomeXchange Consortium via the Proteomics Identification Database (PRIDE) [66] partner repository, with the data set identifier PXD036778. All other methods are described in Supplementary Materials and Methods.

## References

[1] Huang AHC . Oil bodies and oleosins in seeds. Annu Rev Plant Physiol Plant Mol Biol 1992; 43: 177–200. 10.1146/annurev.pp.43.060192.001141

[2] Goold H, Beisson F, Peltier G et al. Microalgal lipid droplets: composition, diversity, biogenesis and functions. Plant Cell Rep 2015; 34: 545–55. 10.1007/s00299-014-1711-7 25433857

[3] Chapman KD, Dyer JM, Mullen RT. Biogenesis and functions of lipid droplets in plants: thematic review series: lipid droplet synthesis and metabolism: from yeast to man. J Lipid Res 2012; 53: 215–26. 10.1194/jlr.R021436 22045929 PMC3269164

[4] Nguyen TB, Olzmann JA. Lipid droplets and lipotoxicity during autophagy. Autophagy 2017; 13: 2002–3. 10.1080/15548627.2017.1359451 28806138 PMC5788477

[5] Xu C, Fan J. Links between autophagy and lipid droplet dynamics. J Exp Bot 2022; 73: 2848–58. 10.1093/jxb/erac003 35560198

[6] Thiam AR, Farese RV, Walther TC. The biophysics and cell biology of lipid droplets. Nat Rev Mol Cell Biol 2013; 14: 775–86. 10.1038/nrm3699 24220094 PMC4526153

[7] Chorlay A, Monticelli L, Veríssimo Ferreira J et al. Membrane asymmetry imposes directionality on lipid droplet emergence from the ER. Dev Cell 2019; 50: 25–42. 10.1016/j.devcel.2019.05.003 31155466

[8] Chorlay A, Thiam AR. Neutral lipids regulate amphipathic helix affinity for model lipid droplets. J Cell Biol 2020; 219: e201907099. 10.1083/jcb.201907099 PMC7147095 32328636

[9] Farese RV Jr, Walther TC. Lipid droplets finally get a little R-E-S-P-E-C-T. Cell 2009; 139: 855–60. 10.1016/j.cell.2009.11.005 19945371 PMC3097139

[10] Szymanski KM, Binns D, Bartz R et al. The lipodystrophy protein seipin is found at endoplasmic reticulum lipid droplet junctions and is important for droplet morphology. Proc Natl Acad Sci USA 2007; 104: 20890–5. 10.1073/pnas.0704154104 18093937 PMC2409237

[11] Valm AM, Cohen S, Legant WR et al. Applying systems-level spectral imaging and analysis to reveal the organelle interactome. Nature 2017; 546: 162–7. 10.1038/nature22369 28538724 PMC5536967

[12] de Vries J, Ischebeck T. Ties between stress and lipid droplets pre-date seeds. Trends Plant Sci 2020; 25: 1203–14. 10.1016/j.tplants.2020.07.017 32921563

[13] Dadras A, Fürst-Jansen JMR, Darienko T et al. Environmental gradients reveal stress hubs pre-dating plant terrestrialization. Nat Plants 2023; 9: 1419–38. 10.1038/s41477-023-01491-0 37640935 PMC10505561

[14] Xu N, Zhang SO, Cole RA et al. The FATP1-DGAT2 complex facilitates lipid droplet expansion at the ER-lipid droplet interface. J Cell Biol 2012; 198: 895–911. 10.1083/jcb.201201139 22927462 PMC3432760

[15] Cai Y, Goodman JM, Pyc M et al. Arabidopsis SEIPIN proteins modulate triacylglycerol accumulation and influence lipid droplet proliferation. Plant Cell 2015; 27: 2616–36. 10.1105/tpc.15.00588 26362606 PMC4815042

[16] Adeyo O, Horn PJ, Lee SK et al. The yeast lipin orthologue Pah1p is important for biogenesis of lipid droplets. J Cell Biol 2011; 192: 1043–55. 10.1083/jcb.201010111 21422231 PMC3063132

[17] Horn PJ, James CN, Gidda SK et al. Identification of a new class of lipid droplet-associated proteins in plants. Plant Physiol 2013; 162: 1926–36. 10.1104/pp.113.222455 23821652 PMC3729771

[18] Yang HY, Galea A, Sytnyk V et al. Controlling the size of lipid droplets: lipid and protein factors. Curr Opin Cell Biol 2012; 24: 509–16. 10.1016/j.ceb.2012.05.012 22726586

[19] Pyc M, Cai Y, Gidda SK et al. Arabidopsis lipid droplet-associated protein (LDAP)—interacting protein (LDIP) influences lipid droplet size and neutral lipid homeostasis in both leaves and seeds. Plant J 2017; 92: 1182–201. 29083105 10.1111/tpj.13754

[20] Pyc M, Gidda SK, Seay D et al. LDIP cooperates with SEIPIN and LDAP to facilitate lipid droplet biogenesis in Arabidopsis. Plant Cell 2021; 33: 3076–103. 10.1093/plcell/koab179 34244767 PMC8462815

[21] Chung J, Wu X, Lambert TJ et al. LDAF1 and Seipin form a lipid droplet assembly complex. Dev Cell 2019; 51: 551–63. 10.1016/j.devcel.2019.10.006 31708432 PMC7235935

[22] Greer MS, Cai Y, Gidda SK et al. SEIPIN isoforms interact with the membrane-tethering protein VAP27-1 for lipid droplet formation. Plant Cell 2020; 32: 2932–50. 10.1105/tpc.19.00771 32690719 PMC7474298

[23] Miklaszewska M, Zienkiewicz K, Klugier-Borowska E et al. CALEOSIN 1 interaction with AUTOPHAGY-RELATED PROTEIN 8 facilitates lipid droplet microautophagy in seedlings. Plant Physiol 2023; 193: 2361–80. 10.1093/plphys/kiad471 37619984 PMC10663143

[24] Siaut M, Cuiné S, Cagnon C et al. Oil accumulation in the model green alga Chlamydomonas reinhardtii: characterization, variability between common laboratory strains and relationship with starch reserves. BMC Biotechnol 2011; 11: 7. 10.1186/1472-6750-11-7 PMC3036615 21255402

[25] Yamaoka Y, Shin S, Choi BY et al. The bZIP1 transcription factor regulates lipid remodeling and contributes to ER stress management in Chlamydomonas reinhardtii. Plant Cell 2019; 31: 1127–40. 10.1105/tpc.18.00723 30894460 PMC6533020

[26] Yoon K, Han D, Li Y et al. Phospholipid:diacylglycerol acyltransferase is a multifunctional enzyme involved in membrane lipid turnover and degradation while synthesizing triacylglycerol in the unicellular green microalga Chlamydomonas reinhardtii. Plant Cell 2012; 24: 3708–24. 10.1105/tpc.112.100701 23012436 PMC3480297

[27] Bai F, Yu L, Shi J et al. Long-chain acyl-CoA synthetases activate fatty acids for lipid synthesis, remodeling and energy production in Chlamydomonas. New Phytol 2022; 233: 823–37. 10.1111/nph.17813 34665469

[28] Tsai C-H, Warakanont J, Takeuchi T et al. The protein Compromised Hydrolysis of Triacylglycerols 7 (CHT7) acts as a repressor of cellular quiescence in Chlamydomonas. Proc Natl Acad Sci USA 2014; 111: 15833–8. 10.1073/pnas.1414567111 25313078 PMC4226073

[29] Jang S, Kong F, Lee J et al. CrABCA2 facilitates triacylglycerol accumulation in Chlamydomonas reinhardtii under nitrogen starvation. Mol Cells 2020; 43: 48–57. 31910336 10.14348/molcells.2019.0262 PMC6999713

[30] Kong F, Liang Y, Legeret B et al. Chlamydomonas carries out fatty acid beta-oxidation in ancestral peroxisomes using a bona fide acyl-CoA oxidase. Plant J 2017; 90: 358–71. 10.1111/tpj.13498 28142200

[31] Kong F, Burlacot A, Liang Y et al. Interorganelle communication: peroxisomal MALATE DEHYDROGENASE 2 connects lipid catabolism to photosynthesis through redox coupling in Chlamydomonas. Plant Cell 2018; 30: 1824–47. 10.1105/tpc.18.00361 29997239 PMC6139685

[32] Goold HD, Cuine S, Legeret B et al. Saturating light induces sustained accumulation of oil in plastidal lipid droplets in Chlamydomonas reinhardtii. Plant Physiol 2016; 171: 2406–17. 10.1104/pp.16.00718 27297678 PMC4972293

[33] Kajikawa M, Sawaragi Y, Shinkawa H et al. Algal dual-specificity tyrosine phosphorylation-regulated kinase, triacylglycerol accumulation regulator1, regulates accumulation of triacylglycerol in nitrogen or sulfur deficiency. Plant Physiol 2015; 168: 752–64. 10.1104/pp.15.00319 25922058 PMC4453788

[34] Merchant SS, Kropat J, Liu B et al. TAG, you're it! Chlamydomonas as a reference organism for understanding algal triacylglycerol accumulation. Curr Opin Biotechnol 2012; 23: 352–63. 10.1016/j.copbio.2011.12.001 22209109

[35] Lee J, Yamaoka Y, Kong F et al. The phosphatidylethanolamine-binding protein DTH1 mediates degradation of lipid droplets in Chlamydomonas reinhardtii. Proc Natl Acad Sci USA 2020; 117: 23131–9. 10.1073/pnas.2005600117 32868427 PMC7502771

[36] Moellering ER, Benning C. RNA interference silencing of a major lipid droplet protein affects lipid droplet size in Chlamydomonas reinhardtii. Euk Cell 2010; 9: 97–106. 10.1128/EC.00203-09 PMC2805299 19915074

[37] Nguyen HM, Baudet M, Cuiné S et al. Proteomic profiling of oil bodies isolated from the unicellular green microalga Chlamydomonas reinhardtii: with focus on proteins involved in lipid metabolism. Proteomics 2011; 11: 4266–73. 10.1002/pmic.201100114 21928291

[38] Tsai C-H, Zienkiewicz K, Amstutz CL et al. Dynamics of protein and polar lipid recruitment during lipid droplet assembly in Chlamydomonas reinhardtii. Plant J 2015; 83: 650–60. 10.1111/tpj.12917 26096381

[39] Wang X, Wei H, Mao X et al. Proteomics analysis of lipid droplets from the oleaginous alga Chromochloris zofingiensis reveals novel proteins for lipid metabolism. Genom Proteomics Bioinformatics 2019; 17: 260–72. 10.1016/j.gpb.2019.01.003 PMC6818385 31494267

[40] Riekhof WR, Sears BB, Benning C. Annotation of genes involved in glycerolipid biosynthesis in Chlamydomonas reinhardtii: discovery of the betaine lipid synthase BTA1(Cr). Euk Cell 2005; 4: 242–52. 10.1128/EC.4.2.242-252.2005 PMC549322 15701786

[41] Li X, Zhang R, Patena W et al. An indexed, mapped mutant library enables reverse genetics studies of biological processes in Chlamydomonas reinhardtii. Plant Cell 2016; 28: 367–87. 10.1105/tpc.15.00465 26764374 PMC4790863

[42] Bai F, Yu L, Shi J et al. Long-chain acyl-CoA synthetases activate fatty acids for lipid synthesis, remodeling and energy production in Chlamydomonas. New Phytol 2022; 233: 823–37. 10.1111/nph.17813 34665469

[43] Warakanont J, Tsai C-H, Michel EJS et al. Chloroplast lipid transfer processes in Chlamydomonas reinhardtii involving a TRIGALACTOSYLDIACYLGLYCEROL 2 (TGD2) orthologue. Plant J 2015; 84: 1005–20. 10.1111/tpj.13060 26496373

[44] Zheng H-Q, Chiang-Hsieh Y-F, Chien C-H et al. AlgaePath: comprehensive analysis of metabolic pathways using transcript abundance data from next-generation sequencing in green algae. BMC Genomics 2014; 15: 196. 10.1186/1471-2164-15-196 24628857 PMC4028061

[45] Moellering ER, Benning C. RNA interference silencing of a major lipid droplet protein affects lipid droplet size in Chlamydomonas reinhardtii. Euk Cell 2010; 9: 97–106. 10.1128/EC.00203-09 PMC2805299 19915074

[46] Nguyen HM, Baudet M, Cuiné S et al. Proteomic profiling of oil bodies isolated from the unicellular green microalga Chlamydomonas reinhardtii: with focus on proteins involved in lipid metabolism. Proteomics 2011; 11: 4266–73. 10.1002/pmic.201100114 21928291

[47] Kyte J, Doolittle RF. A simple method for displaying the hydropathic character of a protein. J Mol Biol 1982; 157: 105–32. 10.1016/0022-2836(82)90515-0 7108955

[48] Jumper J, Evans R, Pritzel A et al. Highly accurate protein structure prediction with AlphaFold. Nature 2021; 596: 583–9. 10.1038/s41586-021-03819-2 34265844 PMC8371605

[49] Nagai Y, Aoki J, Sato T et al. An alternative splicing form of phosphatidylserine-specific phospholipase A1 that exhibits lysophosphatidylserine-specific lysophospholipase activity in humans. J Biol Chem 1999; 274: 11053–9. 10.1074/jbc.274.16.11053 10196188

[50] Sandager L, Gustavsson MH, Ståhl U et al. Storage lipid synthesis is non-essential in yeast. J Biol Chem 2002; 277: 6478–82. 10.1074/jbc.M109109200 11741946

[51] Gu X, Cao L, Wu X et al. A lipid bodies-associated galactosyl hydrolase is involved in triacylglycerol biosynthesis and galactolipid turnover in the unicellular green alga Chlamydomonas reinhardtii. Plants (Basel) 2021; 10: 675. 33807496 10.3390/plants10040675 PMC8065580

[52] Chorlay A, Monticelli L, Ferreira JV et al. Membrane asymmetry imposes directionality on lipid droplet emergence from the ER. Dev Cell 2019; 50: 25–42. 10.1016/j.devcel.2019.05.003 31155466

[53] Chorlay A, Santinho A, Thiam AR. Making droplet-embedded vesicles to model cellular lipid droplets. STAR Protoc 2020; 1: 100116. 10.1016/j.xpro.2020.100116 33377012 PMC7757013

[54] Salo VT, Li S, Vihinen H et al. Seipin facilitates triglyceride flow to lipid droplet and counteracts droplet ripening via endoplasmic reticulum contact. Dev Cell 2019; 50: 478–93. 10.1016/j.devcel.2019.05.016 31178403

[55] Lord CC, Thomas G, Brown JM. Mammalian alpha beta hydrolase domain (ABHD) proteins: lipid metabolizing enzymes at the interface of cell signaling and energy metabolism. Biochim Biophys Acta Mol Cell Biol Lipids 2013; 1831: 792–802. 10.1016/j.bbalip.2013.01.002 PMC4765316 23328280

[56] Fuller N, Rand RP. The influence of lysolipids on the spontaneous curvature and bending elasticity of phospholipid membranes. Biophys J 2001; 81: 243–54. 10.1016/S0006-3495(01)75695-0 11423410 PMC1301507

[57] Jouhet J . Importance of the hexagonal lipid phase in biological membrane organization. Front Plant Sci 2013; 4: 494. 10.3389/fpls.2013.00494 24348497 PMC3848315

[58] Khattib A, Musa S, Halabi M et al. Lyso-DGTS lipid derivatives enhance PON1 activities and prevent oxidation of LDL: a structure–activity relationship study. Antioxidants 2022; 11: 2058. 10.3390/antiox11102058 36290781 PMC9598486

[59] Ben M’barek K, Ajjaji D, Chorlay A et al. ER membrane phospholipids and surface tension control cellular lipid droplet formation. Dev Cell 2017; 41: 591–604. 10.1016/j.devcel.2017.05.012 28579322

[60] Chorlay A, Forêt L, Thiam AR. Origin of gradients in lipid density and surface tension between connected lipid droplet and bilayer. Biophys J 2021; 120: 5491–503. 10.1016/j.bpj.2021.11.022 34808099 PMC8715250

[61] Klug RM, Benning C. Two enzymes of diacylglyceryl-O-4′-(*N,N,N*,-trimethyl)homoserine biosynthesis are encoded by btaA and btaB in the purple bacterium Rhodobacter sphaeroides. Proc Natl Acad Sci USA 2001; 98: 5910–5. 10.1073/pnas.101037998 11331765 PMC33312

[62] Murakami H, Nobusawa T, Hori K et al. Betaine lipid is crucial for adapting to low temperature and phosphate deficiency in nannochloropsis. Plant Physiol 2018; 177: 181–93. 10.1104/pp.17.01573 29555786 PMC5933114

[63] Li X, Moellering ER, Liu B et al. A galactoglycerolipid lipase is required for triacylglycerol accumulation and survival following nitrogen deprivation in Chlamydomonas reinhardtii. Plant Cell 2012; 24: 4670–86. 10.1105/tpc.112.105106 23161887 PMC3531859

[64] Young DY, Shachar-Hill Y. Large fluxes of fatty acids from membranes to triacylglycerol and back during N-deprivation and recovery in Chlamydomonas. Plant Physiol 2021; 185: 796–814. 10.1093/plphys/kiaa071 33822218 PMC8133548

[65] Harris EH . The Chlamydomonas Sourcebook: Introduction to Chlamydomonas and Its Laboratory Use: Volume 1. Amsterdam: Elsevier Science, 2009.

[66] Perez-Riverol Y, Csordas A, Bai J et al. The PRIDE database and related tools and resources in 2019: improving support for quantification data. Nucleic Acids Res 2019; 47: D442–50. 10.1093/nar/gky1106 30395289 PMC6323896

[67] Miller R, Wu G, Deshpande RR et al. Changes in transcript abundance in Chlamydomonas reinhardtii following nitrogen deprivation predict diversion of metabolism. Plant Physiol 2010; 154: 1737–52. 10.1104/pp.110.165159 20935180 PMC2996024

